# Extra Supplement—The Nursing Mirror

**Published:** 1890-05-31

**Authors:** 


### "The Hospital" Nursing Mirror.

Being the Extra Nursing Supplement of "The Hospital" Newspaper.

All Contributions for this Supplement should be addressed to the Editor, The Hospital, 140, Strand, London, W.C., and should have the word "Nursing" plainly written in left-hand top corner of the envelope.

### En Passant.

DURING THE PLAGUE.—In Defoe's "Journal of the Plague Year," which has lately been issued in a cheap form, the accounts of the misdeeds of nurses beat even those told by Dickens. Defoe says: "People—that is to say, hired nurses—who attended infected people used them most barbarously, starving them, smothering them, or by other wicked means hastening their end—that is to say, murdering them." Again: "They did tell me, indeed, of a nurse in one place that laid a wet cloth upon the face of a dying patient, and so put an end to his life." Defoe says he does not believe all these tales, but that he knows that many of the nurses were thieves, and "some of them were publicly whipped for it, when perhaps they ought rather to have been hanged for examples."

REGISTRATION OF MIDWIVES.—So Mr. Bradlaugh employed his tongue to delay the Bill for the registration of midwives, the debate on which had to be adjourned, and the second reading of which has, therefore, not yet taken place. Mr. Bradlaugh's point is that we legislate too much—that our government is too grandmotherly. We wish it was just grandmotherly enough to know that the lives of mothers and infants should be protected. It is grandfatherly enough to say that when Mr. Bradlaugh was so ill a short time since, only a man skilled in medicine should attend and doctor him; but when our mothers approach their hour of labour, Government and Mr. Bradlaugh say that any old woman, no matter how ignorant, shall work her will on anguished woman and wailing babe. Oh! shame, that because legislation is in the hands of men, they neglect to protect woman in her greatest hour of need!

A HOME OF REST.—Truly nurses ought to be most grateful for all the kind thoughts which the public have for them just now; yet some of us would rather have a just wage and a better position than have a convalescent home provided as is done for the waifs and strays of Whitechapel. How it makes a nurse's ears burn when a kindly-intentioned speaker dilates on the lowness of a nurse's wages, and practically begs in the name of nurses as a profession! Still, since it is a fact that many nurses now only earn £20 a year, perhaps the Home of Rest may be a welcome charity for some; but considering the glorious news we hear daily of the juster pay and appreciation of nurses by hospitals and institutions, we hope the day will come when not charity, but self-help will provide pleasant holidays for those who need them most. Has not the Hospital for Sick Children, Great Ormond Street, just adopted the co-operative system for its private nurses? This is far better news than the starting of a charity home. It was amusing at the meeting to note the ignorance, real or affected, of all the speakers on the point of the many holiday homes already existing for nurses. They had none of them ever heard of the Brassey Home, or Sister Mary's Home, at Folkestone, or any other of the half dozen homes of which nurses know. The Bishop of Ripon in speaking at the meeting, wisely advocated tours for nurses, as, on the whole, more restful and changeful than institution life, of which they have too much while on duty. This is a point which will ever debar the larger proportion of nurses from patronising a holiday home; during a holiday they want to break free from nurses and institutions and perpetual talk about cases; they want a mental change as well as a bodily one.

SHORT ITEMS.—Mr. William Rathbone, M.P., has written a history of "District Nursing," which is dedicated to the Queen, and contains an introduction by Miss Florence Nightingale.—We have had two letters about a notice of the death of Miss Nightingale which appeared in a small paper last week. Of course the statement was utterly wrong; it was Miss Nightingale's sister who died. Our correspondents must expect to be misled if they read such papers.—The report of the training school for nurses in connection with the Hospital for Sick Children, San Francisco, is pleasant reading. The matron is Miss E. A. Hurd, who trained in New York, and there are twenty-three pupil nurses.—"Edith's Summer Rest" is the name given to a holiday home for nurses lately established in America. The charge is from four to five dollars a-week, a higher charge than is made by similar homes in England.—Lord Hartington's meeting at Devonshire House in aid of the Children's Country Holidays' Fund was a great success. Over £200 has been transmitted to the treasurer, the Hon. Alfred Lyttelton, 10, Buckingham Street, Strand, W.C.—The Hospitals Association annual meeting will be held on Wednesday next, June 4, at five p.m., at the Middlesex Hospital, Berners Street, Oxford Street, under the presidency of Dr. J. S. Bristowe, F.R.S.

HAMMERSMITH NURSING ASSOCIATION. — An interesting meeting was held on the 20th inst. in the beautiful studio of Mr. W. B. Richmond, at Hammersmith, in aid of the District Nursing Association in that neighbourhood, which association has just begun work. The speakers were Mrs. Garrett Anderson, M.D., Miss Wood (late superintendent, Great Ormond Street Hospital), Mr. Warrington Howard, etc. They pointed out the many benefits—moral, medical, and economical—which a trained nurse of a higher class brings into the homes of the poor; how her example and teaching illustrate the beauty of health and cleanliness, and enforce the sanitary laws of the country; how her daily visits can carry on and complete the treatment begun in the hospital—in many cases, indeed, prevent the necessity of indoor treatment, and so save expense to the already overcrowded hospitals; how she can alleviate by her careful dressing of an incurable wound, or attention to some chronic disease, the sufferings and discomfort of those cases which no hospital admits; how her wise advice prevents the spread of infectious disease and saves many a home from being broken up. We who, even in our comfortable homes, know the trial and disturbance which sickness makes in a household, can imagine the beautiful effects of a daily visit from an intelligent and tender woman, who will "put things straight" in, perhaps, the one room, direct the well-meaning but ignorant efforts of the "next door" neighbour, and leave the patient clean and easy to bear her sickness, and will see that every one of the doctor's directions are carefully carried out. We hope ladies will offer themselves for this branch of charitable work, seeing how direct and complete a work it is. Two years' training at a hospital, six months in district nursing, are considered necessary by the council of the society. And funds, we hope, will come in, for though this is a work that should, above all others, have local support, as it benefits, directly or indirectly, every inhabitant, Hammersmith, though a large, is not a rich neighbourhood; and we trust friends in other districts will come to its aid. The office of the association is at 23, Bridge Road, close to the railway stations and tram line from the western suburbs. Here the nurse can be applied for by the medical men, ministers, or by the poor themselves. The hon. secretary will give every information and receive subscriptions.

### The History and Capabilities of Herbal Simples.

By W. T. Fernie, M.D.

(*Continued from page* 90.)

### X.—BROOM.

Presently, when our English heaths and commons begin to burgeon out into a brilliant luxuriance of yellow and green, many a happy bee will embark in the sunshine for a merry trip on a golden galleon of broom blossom. We may easily watch him alight on the deck of the floral craft, saluting its showy upright petal, which is aptly named the Standard. Beneath is a hollow hold, and within the hull lies a rich store of precious pollen as merchandise. Lateral petals form wings to the sea bird, and are skilfully locked to its sides by cunning well-fitted springs. The stamens lie extended in the keel as cargo, with their gold-dusted ends in the prow. Bounce comes the blithe insect with a lively jump on the quarter deck, and straightway the side springs are set free from their haspings. Forthwith the vessel bounds upwards, and from its open hatchways projects a cloud of diamond dust against the body of the welcome intruder. Enriched with his fertilising burden the glad creature rides awhile triumphant on the breezy main, then hies itself off to visit another bark of the same flowery flotilla, where the pistil is ripe to be fecundated, but where the stamens, like merry dogs, have already had their day. Let any of our readers who may care to repeat for themselves this pretty proceeding impersonate a busy bee on a bright spring morning in the open country where the broom flourishes; and pounce with the end of a pencil on the deck of one of its full blown flowers at the prow. The good ship will at once respond to this mimic "open sesame," and will shoot from its granary a cloud of wealthy pollen as prolific as the golden shower of the fabled and fruitful Danae.

The broom, or link (Cytisus scoparius), grows abundantly as a shrub on open places in our rural districts. It belongs to the Leguminous order of plants, which includes the peas and the vetches, and it formerly bore the name of Planta genista, which has now become historic. Geoffrey of Anjou, we may remember, was married to Matilda, the daughter of Henry the First. This Geoffrey, when encamped on a heath, before going into battle, plucked a golden spray of broom, and fixed it in his helmet. Thus adorned the young warrior entered the field, and was conspicuous throughout the strife by the bright blossoms on his crest. On this account he afterwards took the name of Plantagenet, and transmitted the same to his descendants, who bore it from the time of Henry the Second to that of humpbacked Richard.

A broom pod is figured in the Manual of Heraldry as worn by our second King Henry; and Ingoldsby says, when talking about his bonnet:—

"With a great sprig of broom which he wore as a badge in it,
He was named from this circumstance Henry Plantagenet."

The stalks of the broom, and especially the young twigs are purgative, and act powerfully on the kidneys. They contain chemically an acid principle—scoparin; and an alkaloid, sparteine. For medicinal purposes the terminal twigs are employed, whether fresh or dried, to make a decoction. This is very useful in dropsy from defective action of the heart, but should not be given where congestion of the lungs is present. Half an ounce by weight of the fresh tops should be boiled down in a pint of water to half this quantity, and a wineglassful may be given as a dose every four or six hours. Shepherds believe the broom to be narcotic because their sheep become stupefied or excited when by chance compelled to eat it. The flower buds, when pickled in vinegar, are sometimes used for capers; and the young tender branches are mixed with hops for brewing. It has long been said that witches delight to ride on the broom when represented as its product, a besom. And in Holland, if a vessel lying in dock has a broom tied to the top of its mast, this advertises it as in search of a new owner. Hence has arisen the saying about a woman when seeking another husband, "Zij steetkt dem bezem"—she hangs out the broom. There is a tradition in Suffolk and Sussex which implies that if the yellow broom is brought into a house in the month of May death will come to that house before the year is out. "If you sweep the house with broom in May, You'll sweep the head of the house away." The generic name of the plant—Scoparius—is derived from the Latin—Scopa—a besom: and the prefix, cytisus, from the name of a Greek island where the broom abounded.

For most forms of *chronic* dropsy a compound decoction of broom may be given with great benefit. To make this, broomtops and dandelion roots, of each half-an-ounce, should be boiled in a pint-and-a-half of water down to a pint, and towards the last, half-an-ounce of bruised juniper berries should be added. When cold, the decoction should be strained, and a wineglassful may be given for each dose. "That worthy Prince of famous memory, Henry VIII., King of England, was wont to drinke the distilled water of broome floures against surfets, and diseases therefrom arising."

### American Training Schools for Nurses.

Jonathan is a young fellow of a go-ahead nature, as we all know. Show him some new thing in science, commerce, or practical politics, and if there is good stuff in it, he will be all on the work in a moment. No letting the grass grow under your feet for him, whatever sleepy old John Bull may do. Consequently, we are not surprised to learn how rapid has been the growth of American training schools for nurses, since the days when the first of their kind was started in New York city, in connection with the Bellevue Hospital. Its *raison d'être*, so Jonathan learned, was the education of a body of skilled nurses, who would not only serve the hospitals usefully and intelligently, but to whom could also be entrusted the care of the sick in their own homes. "A capital plan, and one that should be largely extended," said Jonathan, who is nothing if not prompt and practical. That was in 1873. Within the year a second school was opened in Boston, and a third in New Haven, Connecticut; and in this year of grace, 1890, there are no less than thirty-three scattered over the States. We hear of one in Columbia, one in 'Frisco, three in Boston, three in Philadelphia, four in Brooklyn, and so forth—the pioneer city, New York, heading the list with five schools, and making provision for 229 pupils. The excellence of the training received by the pupils in this city, and elsewhere, is testified to by the large and increasing demand for their services as private nurses, and also as superintendents or assistant-superintendents of hospitals. At present this demand largely exceeds the supply, as is shown by the reports of the schools. With regard to the candidates for admission to the schools,—as far, at any rate, as the larger and better known ones are concerned—the position is reversed, the number of applications being far more numerous than the vacancies to be filled up. During one year 386 women applied for admission to the Bellevue training school, while only 47 could be accepted. As a good "common school" education is essential for admission, and as preference is always given to applicants of superior acquirements and general attainments, American nursing should reach, and doubtless does reach, a high standard.

The pupils go through courses of lectures on hygiene, physiology, anatomy, obstetrics, and the principles of nursing and care of the sick; and periodical examinations test the progress of each student, and her fitness for the work on which she has entered. During the first year her practical work is confined entirely to nursing in the hospitals; but after entering upon her second year she may be sent to attend private cases, at the discretion of the superintendent.

As to age of admission, most of the schools take pupils from twenty to forty years of age, but some do not take any under twenty-five or over thirty-five. In nearly all, the number of years required for the full course of training is two; in three it is one and a half; and in the Philadelphia Lying-in School it is given as only one. In four, at least, there are no instructors of the sterner sex; one of these, the Boston City Hospital Training School, having no less than fourteen lady lecturers. At the school in connection with the San Francisco Children's Hospital, again, there are ten female and only three male instructors; but, on the other hand, many keep to the "good old ways," and give the greater part of their work to the sex which, until quite lately, has had a prescriptive right to it, the respective numbers of male and female teachers in all the schools together being 162 and 80.

We may mention here that one out of these thirty-three schools is for male nurses only; it is situated in New York city, and has now been at work for two or three years. The Bellevue Hospital, also, has decided to open a training school for male nurses. A handsome building has lately been erected in the hospital grounds for this purpose through the generosity of Mr. D. D. Mills, of New York; and by this time it is probably in full working order. Another training school, in connection with the McLean Lunatic Asylum in Somerville, Massachusetts, takes male as well as female pupils, some eighty or ninety in all.

With regard to the salaries paid to pupils, there is, as might be expected, a good deal of variation. In two schools—the Washington and the "Brooklyn Maternity," no salary is given; and in the "Hospital of the Good Shepherd," Syracuse, N.Y., and at Detroit, Michigan, a pupil receives only fifteen pounds for her first year. But the greater number of the schools seem to give from eight to ten dollars a month during the first year, or, in other words, from about twenty to five-and-twenty pounds; while in the second year there will be an increase of two or three dollars per month. Those who contend for the equalisation of women's wages with men's will scarcely be pleased to hear that the pupils in the Male Nurses' Training School are paid thirty pounds for the first year, and about thirty-eight for the second—considerably more than the majority of lady pupils get; and that at the lunatic asylum mentioned above, men are paid nearly double as much as women. On the other hand, excluding this asylum school, in which the payment is naturally higher than in others, the very highest salary which we have come upon is that of the New Haven School, for women only, which is given as 182 dollars per annum, or about thirty-eight pounds. Nothing is said about this sum being obtainable during the second year only; and lady nurses may be excused for noting, with perhaps the least tinge of mischievous malice, that it is only during their second year that male nurses are accounted worthy to receive as much as this, and wondering whether, in spite of the higher scale of payment which is considered to be their due, the poor things do not at first prove too hopelessly "unhandy" to receive the high salary which a few fortunate members of the other sex spring to at one jump.

In comparing these salaries with those paid in our own training schools, we must of course bear in mind that money will not go as far in America—or, at any rate, in the part of America with which we are now concerned—as it does here. Our readers may remember that a nurse writing from Albany, whose letter was published some weeks ago in the Nursing Mirror, complained that the board, washing, and lodging of an American private nurse cost her about four times as much as they would in England. Perhaps she hardly expected, in making this statement, to be taken *au grand sérieux;* at any rate, we will not do so, for, dear me! we all like to have our heads now and again, and to be tied down to speak "the truth, the whole truth, and nothing but the truth," with never a spice of petulant or light-hearted exaggeration, would take all the flavour and the zest from life. But another statement of hers—that she was much better off in England with thirty pounds per annum than in America with forty-five, is really not so far from the mark. Substitute "a little better off," and we fancy you have a very fair statement of the difference in the purchasing power of money in England and America.

By far the largest training school in the States is the "Boston City Hospital," which has 123 students, all instructed, as was mentioned above, by women. To it and to the Bellevue, New York (as being the first of its kind), special interest attaches; and although the Bellevue has only about a third as many pupils (leaving out of account the new branch for male nurses, of which we have not particulars to hand), it evidently occupies a high position, as is seen by the large number of candidates for every vacancy. The superintendent reports that applications for admission have even arrived from Persia and Japan; and a movement has been set on foot to establish a school in India for native women, under the direction of trained nurses and physicians.

Altogether, American nurses seem to have a wide and honourable career before them. To use a vulgar phrase, Jonathan "knows what's what"; and seeing that one of the greatest blessings civilisation can bestow is an educated, trained, and, we may add, refined woman to attend on the bed of sickness or of death, he straightway proceeds to evolve her.

### A Letter from South Africa.

Carnarvon Hospital, Kimberley, March 8th, 1890.

Nurse B. writes: "This hospital stands in its own compound; there is a drive up to the hospital, with large beds of flowers and tropical plants on either side of the drive. The Caffir wards are at the back, quite separate from the European wards of the hospital. There are a great many pay wards. Separate room patients pay 16s. a day, stimulants extra; two patients in a room, 10s. a day; three or four in a room, 5s. a day. As far as I can learn at present, the Sisters of Bloafontein undertake the nursing of the hospital. Government allows so much, and each Caffir who is employed by Government, or who works in the mines has 2s. monthly taken off his pay, 1s. for the hospital and 1s. for the Government. There are a great many contributions given to the hospital. The nurses have a nice home in the grounds—a bed-room to themselves and a general sitting-room. Our compound is lovely with flowers and shrubs; a few tropical, but a great many English flowers. There are two resident doctors—Dr. Callander and Dr. Simmonds—and a dispenser. There is a surgical ward, medical ward, and children's ward. There are seven ward nurses, who take charge of so many wards, and have staff nurses under them, and they in turn have probationers under them. There are about fifty nurses in all. Caffir boys do all the rough work—all the sweeping and cleaning, and helping with the meals, and help with the men patients. There is a large operating-room and porter's room. The rooms are all on the ground floor. All the Caffir men are called boys. There is a large dining-room in the hospital, where Sister Henrietta, the head of the hospital, and all the nurses have meals. Breakfast at seven; meat, bread, butter, eggs, bacon, porridge, clotted cream, and tea; no conversation is allowed at breakfast. At half-past seven the day nurses are in their wards and the night nurses leave, and, after giving their report to the Sister, have breakfast at eight. Lunch at half-past ten. Dinner at two; meat, three kinds of fowls, vegetables, pudding or pies, and fruit, milk, or two ounces of pontac—an African wine—beer, or porter to those who are ordered by the doctor to take it. Tea at seven, and supper at nine. Quarter to nine, service in the chapel of the hospital; prayers read by Sister Henrietta. The day nurses are on duty from half-past seven to nine, the night nine to half-past seven. At night there is a good meal at twelve, and tea at four a.m. So many Caffir boys are on night duty to assist the nurses. The nurses have two hours off duty to themselves during the day; from half-past one to half-past eight twice a month; and a month's holiday in the year. The nurses are most kindly cared for, and treated most courteously by the doctors. There are no students, so the probationers are well taught in dressing cases. There is a great deal of typhoid, pneumonia, and malaria fever, and a great many accidents from the mines, and eye diseases, also D.T. cases, which are very bad, and a keeper is constantly employed. There are seven out-nurses, who are almost always busy. I have a great many massage cases, and am never idle. One case I go to at eight in the evening for an hour's massage; the roads are simply frightful, great stones, deep drains to get across, and very rough walking. When I return the cook (who is a Zulu boy) comes back with me. He talks some English, and I have picked up a few words of Zulu. He calls me Dr. Missis—the Caffirs always say missis. The convicts work in the grounds of the hospital, and a most imposing looking officer guards them. I hope these first impressions of an African hospital will interest your readers.

### Everybody's Opinion.

### THE CURE OF DRUNKARDS.

C. M. A. writes: The suggestion was made on Everybody's Page in The Hospital of May 10th that strong newly-made coffee, without milk or sugar, might be used in temperance work, and help a drunkard to fight against the craving for spirits which besets him. The remedy was tried with success some years ago in the case of a poor woman well known to the present writer. A lady who took great interest in her welfare plied her with strong black coffee whenever the desire for drink came on, and continued to do so until the cure was effected. The woman settled down into a most respectable member of society.

### Appointments.

Devon and Exeter Hospital.—Miss Catherine Miller, who trained at the London Hospital from June, 1882, to June, 1884, and has since been Sister at the R. N. Hospital at Haslar, has been appointed Lady Superintendent of nurses at Exeter Hospital.

Cheshunt Cottage Hospital.—Nurse Louisa Cullip, who trained at the London Hospital from 1886 to 1888, and has since been on the private staff of the London, has been appointed Nurse-Matron of this Cottage Hospital.

### Notes and Queries.

### Answers.

*Matron.*—There is a Red Cross Nursing Association at Dublin, of which particulars can be had by applying to the Lady Superior. We know of no other in England, only the Red Cross being the badge of the Army Ambulance Corps. This corps is sometimes spoken of as the Red Cross.

*One who Would Climb.*—We quite agree with you; the subject is treated in the *Hospital Annual* which is now in the press. Still, if you have a large experience in nursing you will be able to "climb," even though you don't possess a certificate.

*L. A. D.*—A district nurse ought not to be treated as a domestic servant. Your chemist's diploma will be useful to you in India. You need not be ashamed to draw your salary if you do all that is demanded of you in return.

### Vacancies.

The following vacancies are announced:

### Matron.

Jessop Hospital, Sheffield; £50.
Woolwich Cottage Hospital; £40.
Kendal Hospital; £45.

### Night Superintendent.

Bristol Royal Infirmary; £40.

### Sister.

Royal Albert Hospital, Devonport.

### Nurses.

Aberdeen Poorhouse; £22.

Arbroath Poorhouse; £30.

Bishop's Stortford Nursing Institution; £25.

Blackheath Institution.

Brighton Children's Hospital; £20.

Bromwich District Hospital; £25.

Canterbury Hospital (night); £24.

Cheltenham General Hospital.

Central London Throat and Ear Hospital; £14.

Church Deaconesses' House, Chester; £24.

Frome Nurses' Home; £22.

General Nursing Institute.

Hanover Institution; £25.

Hollond Institution, Nice.

Huddersfield Nurses' Home.

Jersey Institute; £25.

Leeds Nurses' Institution; £25.

Levick Institution, Paris.

Middlesboro' Nurses' Home; £25.

North-West London Hospital (night); £24.

Newcastle Infirmary; £20.

Pembrokeshire Infirmary.

Pendlebury Hospital for Children; £20.

St. Helena Home, N.W.

St. Peter's Institute, Bedford; £27.

St. Saviour's Infirmary; £20.

Surbiton Institution.

Weston-super-Mare Institute.

Wirral Children's Hospital.

Woolwich Cottage Hospital; £20.

### District Nurses.

East London Nursing Society.

Hulme Nursing Institution; £25.

Jersey Institution; £25.

Queen Victoria's Institute, Edinburgh.

### Probationers.

Bradford Fever Hospital; £12.

Bolton Infirmary.

General Infirmary, Hertford; £12.

North-West London Hospital; £8.

Pembrokeshire Infirmary.

West Norfolk and Lynn Hospital.

Weston-super-Mare Hospital.

Wisbech Hospital.

### Notice.

"NURSE JOYCE."

The Second Part of this story will appear next week.

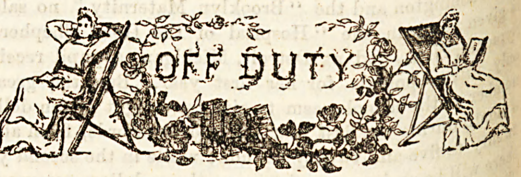

### Amusements and Relaxation.

**N.B.—New Word Competition Commenced April 5th, 1890, ends June 28th, 1890.**

Proper names, abbreviations, foreign words, words of less than four letters, and repetitions are barred; plurals, and past and present participles of verbs, are allowed. Nuttall's *Standard* dictionary only to be used.

The word for dissection for this, the NINTH week of the quarter, being

"CLARENCE."

| Names. | May 22nd. | Totals. | Names. | May 22nd. | Totals. |
|---|---|---|---|---|---|
| Adeline | — | 149 | Agamemnon | 49 | 267 |
| Patience | 49 | 273 | G. E. J. C. | — | 34 |
| Lightowlers | 49 | 257 | S. E. A. | — | 24 |
| Canary | — | 86 | Jenny Wren | 45 | 255 |
| M. E. S. | — | 37 | Multum in parvo | — | 32 |
| Ambition | — | 36 | Ecila | — | 39 |
| M. W. | 49 | 266 | Weta | 25 | 204 |
| Esperance | 49 | 267 | Henri | — | 161 |
| Judy | — | 37 | Holland | 49 | 269 |
| Reldas | 48 | 270 | T. J. | — | 111 |
| Merenda | — | 29 | Nurse Isabel | — | 17 |
| Lucy Locket | — | 40 | Nurse Faith | — | 9 |
| Camellia | — | 30 | E. S. Z. | — | 10 |
| Qu'appelle | 44 | 261 | G. P. | — | 10 |
| Tinie | 49 | 273 | Stumps | — | 33 |
| Nurse Mildred | 27 | 190 | Bolton | — | 8 |
| Coralie | 45 | 265 | Jesmur | — | 53 |
| Gladys | 49 | 269 | Ran | — | 17 |

### Notice to Correspondents.

N.B.—Each paper must be signed by the author with his or her real name and address. A *nom de plume* may be added if the writer does not desire to be referred to by us by his real name. In the case of all prize-winners, however, the real name and address will be published.

Competitors can enter for *all* quarterly competitions, but no competitor may take more than *one first prize* during the year.

